# Commentary: Genomic Analysis Reveals Heterogeneity Between Lesions in Synchronous Primary Right-Sided and Left-Sided Colon Cancer

**DOI:** 10.3389/fmolb.2021.803707

**Published:** 2022-01-21

**Authors:** José Perea, Luis Corchete, Juan L. García, Miguel Urioste, Rogelio González-Sarmiento

**Affiliations:** ^1^ Surgery Department, Fundación Jiménez Díaz University Hospital, Madrid, Spain; ^2^ Fundación Jiménez Díaz Research Institute, Madrid, Spain; ^3^ Hematology Department, Institute of Biomedical Research of Salamanca (IBSAL), Cancer Research Center (CiC-IBMCC, CSIC/USAL), Center for Biomedical Research in Network of Cancer (CIBERONC), University Hospital of Salamanca, Salamanca, Spain; ^4^ Familial Cancer Clinical Unit, Human Cancer Genetics Program, Spanish National Cancer Research Center (CNIO), Madrid, Spain; ^5^ Molecular Medicine Unit, Biomedical Research Institute of Salamanca (IBSAL), Institute of Molecular and Cellular Biology of Cancer (IBMCC), University of Salamanca-USAL-CSIC, Salamanca, Spain

**Keywords:** synchronous colorectal cancer, clonality, metachronous colorectal cancer, heterogeneity, colorectal cancer

We read with interest the recent study by Hu et al., in which they described an approach to elucidate the genomic landscape of synchronous colorectal cancer (SCRC). They used a cohort of paired tumors located in different sites of the colon (right and left colon) and analyzed single nucleotide variation, somatic mutation, and copy number alteration by whole-exome sequencing. The authors finally suggested the heterogeneity between lesions and the polyclonal origin of the paired tumors in the same individual (Hu et al., 2021). They added other previous studies also reporting the heterogeneity and independent genetic origin of SCRC (Cereda et al., 2016; Wang et al., 2018), but without focusing on the location of the paired tumors. On the contrary, to the best of our knowledge, we previously analyzed the largest series of SCRC in exploring clonality, composed by 104 paired SCRCs from 52 consecutive patients without hereditary forms of CRC, using initially a single-nucleotide polymorphism array and a subsequent statistical application to define them according to clonality. Moreover, we used parameters like the mutational concordance and CpG island methylator phenotype (CIMP) status, to confirm our clonality results, and developed a classification according to clonality and paired tumor location, showing clinical correlations. Our results suggested heterogeneity in 64.4% of cases (Figure 1A) but the presence of clonality in 35.6% of cases (Figure 1B) (Perea et al., 2019).

**FIGURE 1:**
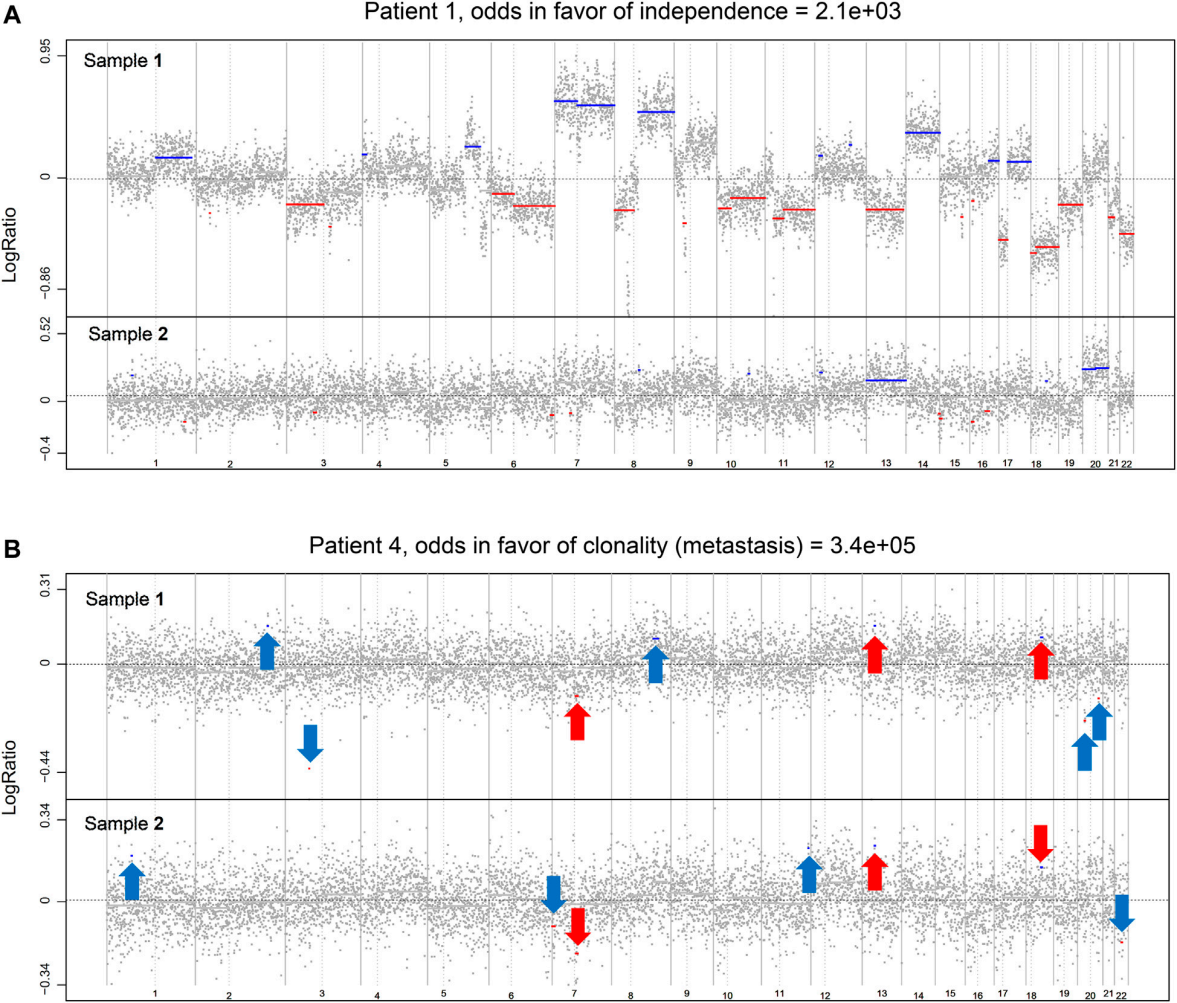
Examples of the Clonality R package genome-wide results based on the likelihood ratio analysis (own data). **(A)** Example of two independent tumors from a CRC patient. **(B)** Example of two clonal tumors from a CRC patient. Red arrows indicate similar copy number (CN) segments. Blue arrows indicate clonal-specific CN segments. Gray dots represent the log_2_ ratios for each genomic position. Chromosome gains are depicted in blue and losses in red.

The importance of the method used to define clonality between paired tumors, multiple primary neoplasms, or metastatic disease, should also be critical. In this regard, most authors analyzed each case independently employing diverse approaches, such as focusing solely on shared mutations between paired tumors, ignoring the information from mutations that occur in one tumor but not the other, evidence that argues against clonal relatedness (Teixeira et al., 2004; Schultheis et al., 2015). Other authors have used the proportion of observed mutations that are shared as the index (Cereda et al., 2016), or assuming that the matched mutations follow a binomial distribution (Bao et al., 2015). Mauguen et al. analyzed the data from all cases collectively to obtain parametric estimates of the proportion of cases in the population that are clonal, and also count heavily on the recognition of the fact that the probabilities of occurrence of the observed mutations are crucially informative, especially for shared mutations (Mauguen et al., 2019). The comparison between the left-sided and right-sided lesions carried out by Hu et al. with SNP signature and tumor mutation burden was performed by a two-tailed paired *t*-test alone, while our results showing clonality were based on a likelihood ratio statistic calculated by the R software package Clonality (Ostrovnaya et al., 2011), which quantifies the odds that the two tumors are clonal. This method uses the copy number profiles from all samples, to determine whether two tumors from the same patient were clonal or origin-independent.

In our previous work (Perea et al., 2019), we classified the location of the paired tumors as monosegmental, when both were in the same colon segment (right or left colon), and pancolonic, when both were distributed all over the entire colon (right and left colon). The distribution of tumors according to the tumor location showed equivalent distribution for both monosegmental and pancolonic cases, with 65.5 and 61% of heterogeneous/polyclonal tumors, respectively. The use of a small cohort is also a limitation, also pointed out by Hu et al., and the need for larger studies with enough cases appears necessary to build a solid definition and approach to define clonal tumors, independently of the type of multiple paired tumors (multiple primary cases or metastasis and primary neoplasm).

Regarding other types of multiple primary colorectal cancers (CRCs), such as metachronous colorectal cancers, recent studies have hypothesized that a small proportion could also be from the same origin, as they showed similar molecular signatures (Backes et al., 2019). An analysis of metachronous CRCs (MCRCs) performed by Backes et al. led the authors to hypothesize that primary tumor cells might be seeded in a new location after biopsy of the primary tumor. In this study, only three cases were considered, and we think that the clonal theory should not be discarded. Taking into account both groups of clonal CRCs regarding both SCRCs and MCRCs, we think that, after subsequent prospective larger studies, the concept of SCRCs and MCRCs could be redefined. Therefore, those cases of multiple primary tumors showing a clonal origin should be clearly differentiated from the others, maybe defined as multiple clonal neoplasms, and the heterogeneous multiple tumors occurring in the same individual may be the ones defined only as multiple primary tumors (synchronous or metachronous, using the concept of temporality, currently the only one used). We also keep in mind the possibility that clonal tumors could be a variety of metastatic disease, together with the diagnostic and therapeutic implications that this would involve.

## References

[B1] Backes Y. Seerden T. C. J. van Gestel R. S. F. E. Kranenburg O. Ubink I. Schiffelers R. M. (2019). Tumor Seeding during Colonoscopy as a Possible Cause for Metachronous Colorectal Cancer. Gastroenterology 157 (5), 1222–1232. 10.1053/j.gastro.2019.07.062 31419435

[B2] Bao L. Messer K. Schwab R. Harismendy O. Pu M. Crain B. (2015). Mutational Profiling Can Establish Clonal or Independent Origin in Synchronous Bilateral Breast and Other Tumors. PLoS ONE 10 (11), e0142487. 10.1371/journal.pone.0142487 26554380 PMC4640562

[B3] Cereda M. Gambardella G. Benedetti L. Iannelli F. Patel D. Basso G. (2016). Patients with Genetically Heterogeneous Synchronous Colorectal Cancer Carry Rare Damaging Germline Mutations in Immune-Related Genes. Nat. Commun. 7, 12072. 10.1038/ncomms12072 27377421 PMC4935966

[B4] Hu H. Zhang Q. Huang R. Gao Z. Yuan Z. Tang Q. (2021). Genomic Analysis Reveals Heterogeneity between Lesions in Synchronous Primary Right-Sided and Left-Sided Colon Cancer. Front. Mol. Biosci. 8 (8), 689466. 10.3389/fmolb.2021.689466 34422903 PMC8371635

[B5] Mauguen A. Seshan V. E. Ostrovnaya I. Begg C. B. (2019). An EM Algorithm to Improve the Estimation of the Probability of Clonal Relatedness of Pairs of Tumors in Cancer Patients. BMC Bioinformatics 20, 555. 10.1186/s12859-019-3148-z 31703552 PMC6839069

[B6] Ostrovnaya I. Seshan V. E. Olshen A. B. Begg C. B. (2011). Clonality: an R Package for Testing Clonal Relatedness of Two Tumors from the Same Patient Based on Their Genomicprofiles. Bioinformatics 27, 1698–1699. 10.1093/bioinformatics/btr267 21546399 PMC3106202

[B7] Perea J. García J. L. Corchete L. Lumbreras E. Arriba M. Rueda D. (2019). Redefining Synchronous Colorectal Cancers Based on Tumor Clonality. Int. J. Cancer 144, 1596–1608. 10.1002/ijc.31761 30151896 PMC6361712

[B8] Schultheis A. M. Ng C. K. Y. De Filippo M. R. Piscuoglio S. Macedo G. S. Gatius S. (2015). Massively Parallel Sequencing-Based Clonality Analysis of Synchronous Endometrioid Endometrial and Ovarian Carcinomas. JNCI.J 108 (6), djv427. 10.1093/jnci/djv427 PMC4909128 26832770

[B9] Teixeira M. R. Ribeiro F. R. Torres L. Pandis N. Andersen J. A. Lothe R. A. (2004). Assessment of Clonal Relationships in Ipsilateral and Bilateral Multiple Breast Carcinomas by Comparative Genomic Hybridisation and Hierarchical Clustering Analysis. Br. J. Cancer 91 (4), 775–782. 10.1038/sj.bjc.6602021 15266323 PMC2364777

[B10] Wang X. Fang H. Cheng Y. Li L. Sun X. Fu T. (2018). The Molecular Landscape of Synchronous Colorectal Cancer Reveals Genetic Heterogeneity. Carcinogenesis 39, 708–718. 10.1093/carcin/bgy040 29546405 PMC5932564

